# The Pathology Lesion Patterns of Podocytopathies: How and why?

**DOI:** 10.3389/fcell.2022.838272

**Published:** 2022-02-24

**Authors:** Fiammetta Ravaglia, Maria Elena Melica, Maria Lucia Angelotti, Letizia De Chiara, Paola Romagnani, Laura Lasagni

**Affiliations:** ^1^ Nephrology and Dialysis Unit, Santo Stefano Hospital, Prato, Italy; ^2^ Department of Experimental and Clinical Biomedical Sciences “Mario Serio”, University of Florence, Florence, Italy; ^3^ Nephrology Unit, Meyer Children’s Hospital, Florence, Italy

**Keywords:** podocytopathies, minimal change disease, focal segmental glomerulosclerosis, diffuse mesangial sclerosis, collapsing glomerulopathy, minimal change, parietal epithelial cells, renal progenitor

## Abstract

Podocytopathies are a group of proteinuric glomerular disorders driven by primary podocyte injury that are associated with a set of lesion patterns observed on kidney biopsy, i.e., minimal changes, focal segmental glomerulosclerosis, diffuse mesangial sclerosis and collapsing glomerulopathy. These unspecific lesion patterns have long been considered as independent disease entities. By contrast, recent evidence from genetics and experimental studies demonstrated that they represent signs of repeated injury and repair attempts. These ongoing processes depend on the type, length, and severity of podocyte injury, as well as on the ability of parietal epithelial cells to drive repair. In this review, we discuss the main pathology patterns of podocytopathies with a focus on the cellular and molecular response of podocytes and parietal epithelial cells.

## Introduction

Podocytes are differentiated epithelial cells whose number is determined shortly before birth as nephrogenesis ceases (Bertram et al., 2011). Podocytes have a limited capacity to complete successful cytokinesis as cell division requires a rearrangement of the podocyte actin cytoskeleton, disrupting the podocyte foot processes (Lasagni et al., 2013). Consequently, direct or indirect podocyte injury, which causes cytoskeleton rearrangement, poses a serious threat to kidney barrier function maintenance, which is reflected in glomerular proteinuria levels.

Podocyte injury can be caused by immunologic, infectious or toxic agents, as well as patient specific characteristics such as obesity or haemodynamic modifications (Kopp et al., 2020). In addition, genetic analysis techniques have broadened the known causes of podocytopathies adding genetic variants to the list. Currently, more than fifty podocyte-expressed genes have been identified as directly linked to podocytopathies as well as syndromal non-podocyte-specific genes, phenocopies with other underlying genetic abnormalities and susceptibility genes (i.e., apolipoprotein L1 (APOL1) variants) leading to a complete revision of carrier risk stratification (Freedman et al., 2014; Warejko et al., 2018; Landini et al., 2020). Podocyte injury can be observed in the setting of all forms of immune complex glomerulonephritis (i.e., lupus nephritis, membranous nephropathy) and in metabolic disorders (diabetes, amyloidosis) and is a key event in chronic kidney disease progression (Kopp et al., 2020). The latter will not be the topic of this review. We will rather focus on disorders that have podocyte as a primary target of injury and that are associated with a variety of lesion patterns that renal pathology struggles to classify (Ahn and Bomback, 2020). These histopathological lesion patterns range from 1) minimal changes (MC), traditionally referred to as “minimal change disease” (MCD) and defined as minimal alterations visible only by ultrastructural analysis; 2) focal segmental glomerulosclerosis (FSGS) where sclerotic lesions are restricted to a subset of the glomeruli; 3) diffuse mesangial sclerosis (DMS) characterized by mesangial matrix increase and podocyte hypertrophy; and lastly 4) collapsing glomerulopathy (CG) which presents as collapse of the glomerular capillaries and hyperplasia of parietal epithelial cells (PECs) migrating to the tuft forming “pseudocrescents” (Barisoni et al., 2007). These glomerular histopathological lesion patterns can be collectively viewed as podocytopathies and their progression to chronic kidney disease is related to the amount of podocyte loss (Kopp et al., 2020). In addition to podocytes and PECs, glomerular endothelial cells and mesangial cells likely contribute to the progression of podocytopathies (Chung et al., 2020). However, as a comprehensive analysis of all the cell types involved in the disease progression is beyond the scope of this review, we will focus our discussion on the PEC-podocyte axis.

Several lines of evidence support the podocyte depletion hypothesis (Kim et al., 2001; Wharram et al., 2005; Fukuda et al., 2012a). In particular, the Wiggins group (Wharram et al., 2005) elegantly showed the consequences of podocyte loss: less than 20% of podocyte loss is associated with a normal glomerulus at light microscopy or with mesangial expansion, whereas loss of more than 20% of podocytes leads to segmental denudation of glomerular basement membrane with consequent adhesions between the Bowman capsule and the glomerular capillary loop. Once the process is initiated sclerosis follows. Segmental sclerosis occurs if podocyte loss is less than 40%, while global sclerosis occurs if podocyte loss exceeds 60% (Wharram et al., 2005).

A subpopulation of PECs endowed with progenitor characteristics can replace, at least to some extent, lost podocytes (Peired et al., 2013; Zhang et al., 2013; Eng et al., 2015; Lasagni et al., 2015; Romoli et al., 2018; Kaverina et al., 2019). However, PECs may also have a detrimental role, as proliferation of activated PECs can also be a crucial determinant of glomerulosclerosis (Lasagni et al., 2015; Eymael et al., 2018).

Collectively, the aim of this review is to: 1) discuss the current concept of histopathological pattern recognition; 2) introduce the importance of PECs in the formation and identification of these peculiar lesions; 3) consider the relationship between PECs and podocytes as an important determinant of disease progression.

## Minimal Changes

### How—Pathology

By definition, glomerular appearance observed by light microscopy in minimal changes is normal, while tubular compartment showed vacuolar, lipoid changes in the proximal tubules defined as lipoid nephrosis (Munk, 1918).

Ultrastructural examination is required to identify the only consistent glomerular pathologic feature of minimal changes, which is simplification of podocyte shape at ultrastructural level without glomerular abnormalities at light microscopy. By electron microscopy, this feature is visualized as effacement of the discrete foot processes and it may be associated to coarsening, i.e., retraction, shortening and widening of foot processes (De Vriese et al., 2018). Foot process simplification and effacement are the earliest morphological patterns of podocyte injury and are clinically associated with highly selective nephrotic-range proteinuria (Vivarelli et al., 2017). Extension of foot process effacement and coarsening is an issue. It has been hypothesized that the surface area extension of capillary loops involved in this process could differentiate the MC lesion pattern from FSGS (De Vriese et al., 2018). Specifically, MC lesion would show widespread foot process effacement and coarsening, involving at least more than half of the capillary loop surface area, while FSGS would show more segmental alterations (Deegens et al., 2008; da Silva et al., 2020) as in maladaptive FSGS increased fluid shear stress is typically a segmental phenomenon (Kriz and Lemley, 2017a). Nevertheless, such an assessment is prone to inaccuracy considering that the ultrastructural analysis is bidimensional and it is limited in the number of glomeruli analyzed (i.e., no more than one or two glomeruli). In addition, no correlation between foot process effacement measured by electron microscopy and proteinuria severity exists, suggesting that this evaluation alone is not sufficient to allow clinical-pathological correlations (Royal et al., 2020).

**FIGURE 1:**
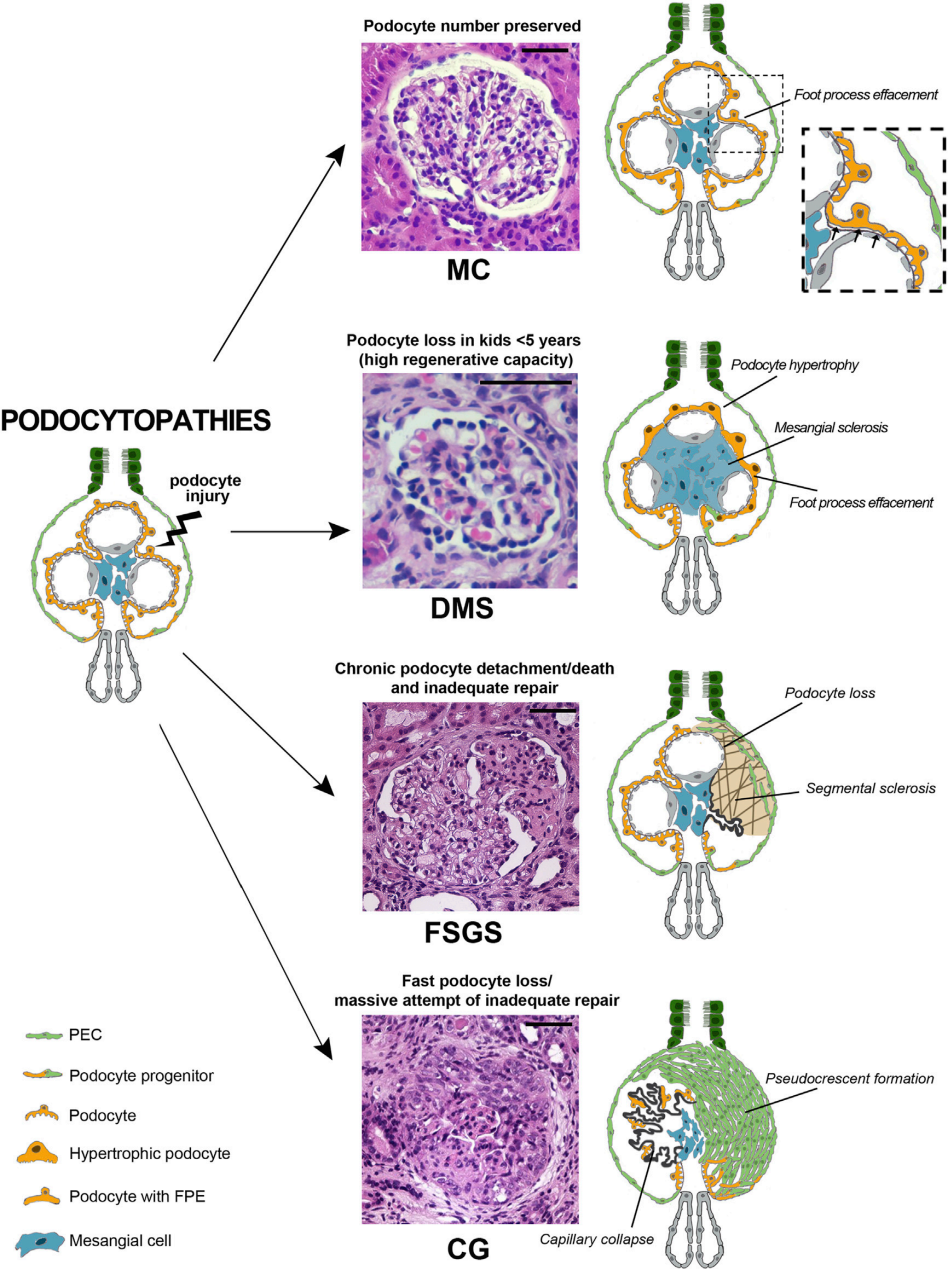
Podocytopathies result from the equilibrium between the nature of injury and the glomerular capacity of repair. When podocyte injury does not determine net cell loss, no changes are present on light microscopy and only foot process effacement is detectable, as in minimal changes (MC). In a setting of fast podocyte loss in kids younger than 5 years old, massive attempt of repair takes place. Immature podocytes are generated and are visible as a halo of hypertrophic podocytes overlying capillary loops as in diffuse mesangial sclerosis (DMS). When severity or chronicity of podocyte injury overcomes the capacity of PECs to replace detached or loss podocytes, glomerular basement membrane denudation triggers an injury cascade. This results in the segmental solidification of the tuft with accumulation of extracellular matrix characteristic of focal segmental glomerulosclerosis (FSGS). Finally, if fast podocyte loss occurs in individuals where the regenerative capacity is inadequate, generation of new podocytes is hampered and proliferating progenitors accumulate in Bowman space in the form of pseudocrescents resulting in collapsing glomerulopathy (CG) (Hematoxylin and eosin stains, magnifications 40x. Bars= 50 μm). (Abbreviations: MC= minimal changes, DMS= diffuse mesangial sclerosis, FSGS= focal segmental glomerulosclerosis, CG= collapsing glomerulopathy, PEC= parietal epithelial cell, FPE= foot process effacement).

Foot process effacement is also associated with microfilament aggregation at the base of epithelial cells and filtration slit distortion, resulting in a reduction in the number of slit diaphragms (Patrakka et al., 2002). This, in turn, causes a displacement of the diaphragms in a ladder-like formation toward the pore apex (Patrakka et al., 2002). In addition, podocytes may show other non-specific structural alterations including hypertrophy, microvillous transformation, formation of vacuoles and the presence of resorption droplets (Royal et al., 2020). However, these alterations do not prevent podocytes from fully covering the glomerular basement membrane. Accordingly, in MC lesions, areas of glomerular basement membrane denudation (Lahdenkari et al., 2004) due to reduction in podocyte density or to net podocyte loss compared to healthy controls do not occur (Szeto et al., 2015) (Figure 1).

**FIGURE 2:**
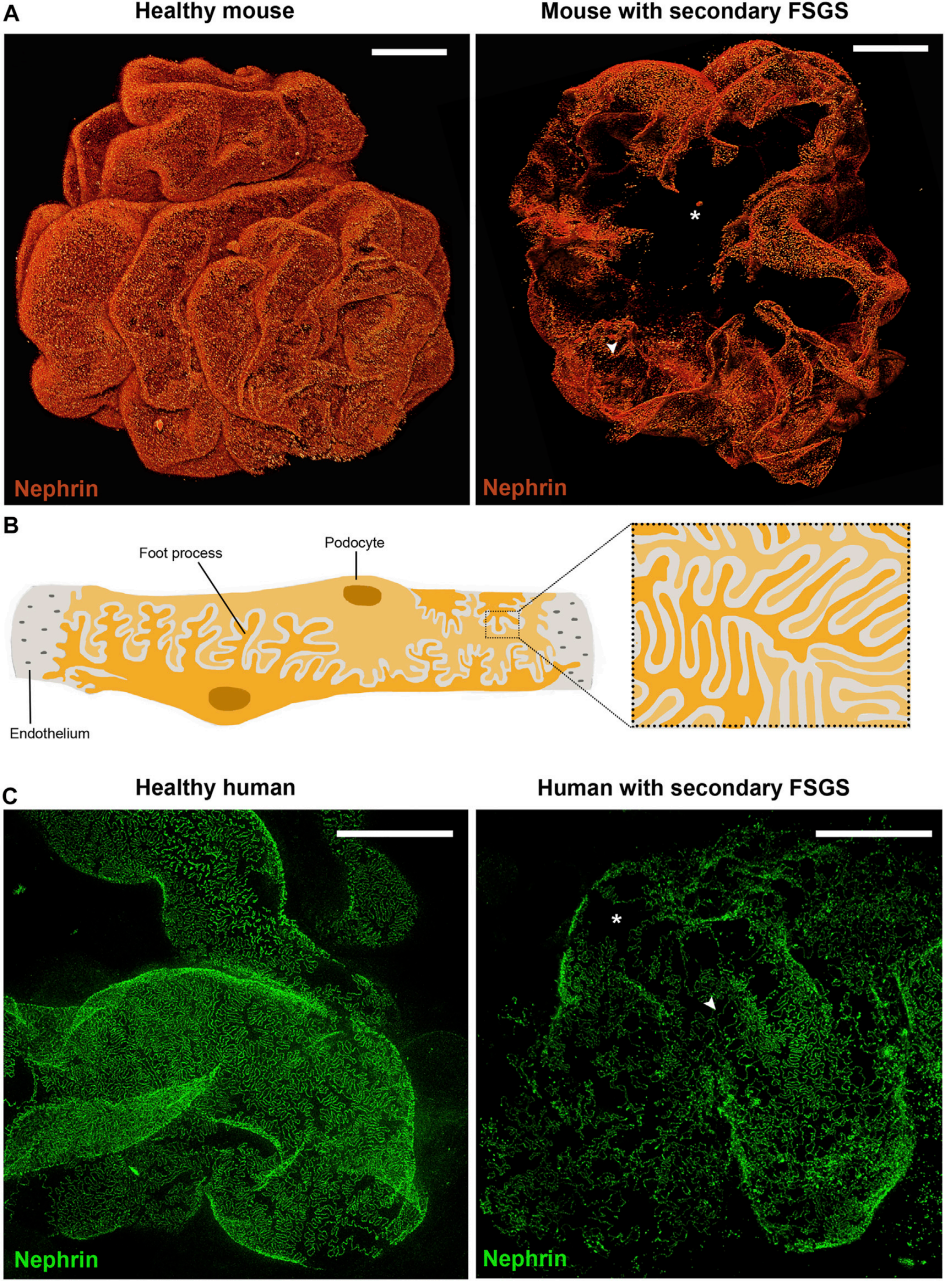
Podocytes are crucial for integrity of the filtration barrier. **(A)** Three-dimensional reconstruction of whole mouse glomeruli stained for nephrin upon optical tissue clearing by using confocal microscopy. The signal represents nephrin protein within the slit diaphragm. Z-series stacks were obtained from 80-μm kidney slices with images collected at 1 μ m intervals. On the left a representative glomerulus from a healthy mouse shows an intact filtration barrier. On the right a representative glomerulus from a mouse with secondary FSGS (obesity- related diabetic mouse, db/db mouse) shows large denudated areas with podocyte loss (asterisk) and foot process effacement (arrowhead). **(B)** Schematic drawing of representative podocytes, with their interdigitating foot processes, wrapped around a glomerular capillary loop. **(C)** Representative images of human podocyte foot processes by using STED-super resolution microscopy upon tissue clearing. Z-series stacks were obtained from 5-μm kidney slices. The green signal represents nephrin protein. On the left a representative image of a normal human kidney obtained from a patient who underwent nephrectomy for localized renal tumor. On the right a representative image of a kidney biopsy obtained from a patient with secondary FSGS, showing denudated areas with podocyte loss (asterisk) and foot process effacement (arrowhead) (Bars= 20 μm)*.*

Finally, immunofluorescence for immunoglobulins and complement fractions on kidney biopsy is generally negative (Vivarelli et al., 2017). However, recent findings of nephrin autoantibodies in a subset of patients with MC lesions at light microscopy and podocyte-associated punctate IgG at immunofluorescence provide support for an autoimmune etiology (Watts et al., 2021). Collectively, the lack of pathological features detectable by light microscopy and immunohistochemistry in biopsies of patients with MC lesions complicates the diagnostic process. Ongoing studies are attempting to identify new biomarkers to predict outcomes or individualize treatments. Nevertheless, podocyte injury at kidney biopsy may be difficult to identify and characterize, mostly due to the focal nature of the damage, inadequate sampling and specific shortcomings linked to traditional techniques, as previously mentioned. Recently, the combination of optical clearing techniques with state-of-the-art microscopy permitted morphometric analysis in thick tissues with a resolution up to nanoscale levels (Angelotti et al., 2021). This technique permits imaging of large tissue areas and to resolve fine structural details at once. Therefore, we can visualize the slit diaphragm 3D, giving direct evidence of structural changes or podocyte loss (Figure 2) (Artelt et al., 2018; Tesch et al., 2021).

### Why—Experimental Evidence

Podocyte foot process effacement is the ultrastructural hallmark of MC lesion, however, the process leading to podocyte effacement is not clear (Purohit et al., 2021). Immunologic dysregulations and modifications of the podocyte are thought to synergize in altering the integrity of the glomerular barrier and therefore determining proteinuria (Purohit et al., 2021). Animal models could potentially provide valuable insights into physiopathology of the MC lesion, but we lack an animal model fitting the specific MC characteristics, i.e., abrupt onset selective nephrotic-range proteinuria, diffuse foot process effacement but no podocytopenia, low rates of disease progression, and steroid sensitivity (Chugh et al., 2012). In fact, all mouse models of chronic and heavy proteinuria eventually develop FSGS following an initial phase with only diffuse podocyte foot process effacement, offering the opportunity to elucidate mechanisms of progression rather than that of acute renal damage. The models most broadly employed by researchers are the Puromycin Aminonucleoside (PAN) model, in which single low-dose injection of toxic agent directed against podocyte molecules induces transient proteinuria and foot process effacement, without inducing podocyte loss (Kim et al., 2001; Pippin et al., 2009) and the diphtheria toxin (DT) model, in which DT injection into rats expressing human diphtheria toxin receptor transgene results in dose-dependent podocyte depletion (Wharram et al., 2005). In particular, in the DT model, less than 20% of podocyte depletion results in mesangial expansion, transient proteinuria, and normal kidney function. The continuum between the MC lesion and FSGS in these models supports the hypothesis that in some patients these two lesion patterns represent different pathology manifestations of the same injury (Maas et al., 2016).

Long before animal models, the possible link between MC and FSGS lesions had already been suggested in the first clinical report of nephrotic syndrome in the 1970s (Habib and Kleinknecht, 1971). Afterwards, identification of FSGS in patients that previously exhibited MC biopsy pattern was reported in children who underwent repeated kidney biopsies during the course of steroid sensitive nephrotic syndrome (Tejani, 1985) and in serial post-transplantation biopsies of patients with FSGS recurrence (Charnaya and Moudgil, 2017), supporting evidence of an evolving process (Maas et al., 2016). During the progression from MC to FSGS lesion, the glomeruli significantly increase their volumes and podocyte hypertrophy appears as a distinguishing feature of FSGS *vs.* MC (Fogo et al., 1990). This would suggest that interventions aimed at regulating glomerular volume and podocyte hypertrophy could represent an effective strategy to sustain podocyte survival and to prevent podocytopenia (Puelles et al., 2019; Banu et al., 2021). However, this adaptive response of podocytes is only sustainable until a threshold is reached, after which the podocyte detaches and progression to FSGS occurs. Podocyte detachment is thought to occur through a substantial increase in the mechanical forces of fluid filtration (Saga et al., 2021). Evaluation of mechanical properties of podocytes and of their response to external mechanical stimuli has shown how circumferential wall tension as well as fluid shear stress play an important role in podocytopathies and progression from MC to FSGS lesion (Kriz and Lemley, 2017b; Embry et al., 2018; Calizo et al., 2019). It is also possible that in MC podocyte loss occurs but it is not detectable either because it does not differ from normal physiological turnover of healthy glomeruli or because the progenitors succeed in replacing lost podocytes. Indeed, many studies reported the PEC capacity to differentiate into mature podocytes after glomerular injury, replacing at least in part the lost podocytes (Peired et al., 2013; Zhang et al., 2013; Eng et al., 2015; Lasagni et al., 2015; Romoli et al., 2018; Kaverina et al., 2019).

If the cause of podocyte injury is reversible, we observe a return to normal of foot processes together with a complete proteinuria remission and a favorable prognosis. In case of irreversible podocyte injury, podocyte loss can still be partially compensated by progenitor replacement (Nagata, 2016; Kopp et al., 2020). When podocyte loss reaches its tipping point (more than 20% of podocytes are lost), PECs are unable to fully compensate for podocyte loss. In such cases, the MC lesion represents the first stage of a condition that will progress towards more severe patterns of injury characterized by more prominent podocyte loss (i.e., FSGS) (Figure 1 and Table 1).

**TABLE 1:** Animal models and clinical evidence supporting the proposed pathomechanisms for development of pathology lesion patterns associated with podocytopathies.

Minimal changes (foot process effacement and podocyte loss lower than 20%)				
		**Evidence**	**Study limitations**	
Minimal changes develops in absence of podocyte loss or for loss lower than 20%	Animal models	Development of FPE and proteinuria following low dose injection of toxic agents and dose dependent podocyte depletion shows normal glomeruli when podocyte loss is absent or limited Kim et al. (2001); Wharram et al. (2005); Pippin et al. (2009); Banu et al. (2021)	After an initial phase with only FPE, FSGS develops in this model	
	Clinical evidence	Normal glomeruli on light microscopy in biopsies of patients	Alteration missing owing to sampling error	
		Absence of podocyte excretion or low levels of podocyte mRNAs in urine of patients with minimal changes Szeto et al. (2015)	Podocytes die but do not detach from GBM	
Minimal changes progress toward focal segmental sclerosis	Animal models	FSGS develops after an initial phase with only FPE in all the animal models with persistent proteinuria Kim et al. (2001); Wharram et al. (2005); Pippin et al. (2009)	NA	
		Podocyte depletion and FSGS development are dose-dependent Kim et al. (2001); Wharram et al. (2005)	NA	
	Clinical evidence	Appearance of FSGS in second biopsies in patients previously diagnosed with MC Tejani, (1985) and in post-transplant biopsies of patients with FSGS recurrence after diagnosis of MC Maas et al. (2016); Charnaya and Moudgil, (2017)	FSGS is missed owing to sampling error	
Diffuse Mesangial Sclerosis (podocytes loss in the setting of high podocyte replacement-early childhood)				
		**Evidence**	**Study limitations**	
Diffuse mesangial sclerosis develops following podocyte loss	Animal models	Mesangial expansion is the first evidence of podocyte loss by 20% or less Wharram et al. (2005)	Podocyte loss induced in adult rats do not reproduce human DMS	
	Clinical evidence	Podocyte excretion in urine of patients with DMS Ikezumi et al. (2014)	NA	
High capacity to generate new podocyte by PECs during childhood	Animal models	Generation of podocytes from PECs during kidney development before birth Wanner et al. (2014) and during postnatal glomerular growth Appel et al. (2009)		Lineage tracing of PECs started before birth Wanner et al. (2014)
		Generation of 10% of podocytes from genetically tagged PECs during postnatal glomerular growth Lasagni et al. (2015)		NA
	Clinical evidence	Less-differentiated podocyte phenotype and increased expression of the PEC progenitor marker Pax2 in glomeruli of patients with DMS Yang et al. (1999)		NA
		Proliferating cells positive for claudin-1 in glomeruli of children with DMS Ikezumi et al. (2014)		NA
FSGS (chronic severe podocyte loss and PEC activation with inadequate podocyte replacement)				
		**Evidence**		**Study limitations**
FSGS lesions develop following chronic severe podocyte loss and PECs activation but inefficient differentiation with inadequate podocyte replacement	Animal models	FSGS develops for podocyte depletion between 21 and 40% Wharram et al. (2005) or for chronic proteinuria Kim et al. (2001); Pippin et al. (2009), and results in PEC activation and glomerulosclerosis Puelles et al. (2019)		NA
		Podocyte depletion and FSGS development are dose-dependent Kim et al. (2001); Wharram et al. (2005); Pippin et al. (2009); Fukuda et al. (2012b)		NA
		CD44 and CD9 expression in PECs during FSGS Okamoto et al. (2013); Lazareth et al. (2019)		NA
		Lesions in FSGS are generated by genetically tagged PECs Lasagni et al. (2015); Romoli et al. (2018)		NA
		Generation of podocytes from PECs in FSGS Eng et al. (2015); Kaverina et al. (2019) and during aging Kaverina et al. (2020)		NA
		Pharmacological treatment induces remission of proteinuria and increase in podocyte number enhancing generation of podocytes by genetically labelled PECs Lasagni et al. (2015); Romoli et al. (2018)		NA
		Increased podocyte density and/or in number of PEC progenitors in response to pharmacological treatment Peired et al. (2013); Zhang et al. (2013); Zhang et al. (2012); Hudkins et al. (2020); Zhang et al. (2015); Motrapu et al. (2020)		No lineage tracing to determine the origin of new podocytes
	Clinical evidence	Reduced number of podocytes in biopsies Motrapu et al. (2020)		Semi-quantitative podocyte counting
		Presence of podocytes and podocyte mRNA in urine of patients affected by FSGS Szeto et al. (2015)		NA
		CD44 and CD9 expression in glomeruli of FSGS patients Lazareth et al. (2019); Fatima et al. (2012)		NA
		Markers of PEC progenitor in FSGS lesions from biopsies of patients Smeets et al. (2014); Dijkman et al. (2005); Smeets et al. (2009)		NA
		Increase in podocyte number, remission and regression of functional parameters of CKD in patients with diabetic and nondiabetic nephropathies Fioretto et al. (1998); Remuzzi et al. (2006); Takahashi et al. (2007); Cortinovis et al. (2016); Boffa et al. (2003); Ma et al. (2005); Fogo, (2001); Yang and Fogo, (2014)		NA
Collapsing glomerulopathy (severe podocyte loss and dysregulated PEC/RPC activation)				
		**Evidence**		**Study limitations**
Collapsing glomerulopathy develops following severe podocyte loss and pseudocrescents originate from the proliferation of PECs progenitors	Animal models	Global glomerulosclerosis for podocyte depletion >40% Wharram et al. (2005)		NA
		Extensive podocyte loss and simultaneous PEC hyperplasia in collapsing FSGS Suzuki et al. (2009)		NA
		Podocyte loss triggers the activation of a distinct PEC subpopulation Pace et al. (2021)		NA
		PEC to podocyte differentiation in HIV nephropathy Dai et al. (2017)		No lineage tracing to determine the origin of new podocytes
		Presence of cells expressing PEC and podocyte markers in glomeruli of HIV transgenic mice expressing APOL1 Kumar et al. (2018)		
	Clinical evidence	Expression of PEC progenitor markers in proliferating cells of pseudocrescent Lazareth et al. (2019); Dijkman et al. (2006); Smeets et al. (2009); Smeets et al. (2004)		NA
		Presence of PEC expressing PEC and podocyte markers in glomeruli of patients with HIVAN Kumar et al. (2018)		NA

(Abbreviations: FPE= foot process effacement, FSGS= focal segmental glomerulosclerosis, GBM= glomerular basement membrane, MC= minimal changes, DMS= diffuse mesangial sclerosis, PEC= parietal epithelial cell, CKD= chronic kidney disease, APOL1= apolipoprotein L1, HIVAN= HIV associated nephropathy).

## Diffuse Mesangial Sclerosis

### How—Pathology

DMS is found in children younger than 5 years old with nephrotic syndrome progressing to end stage kidney disease during childhood (Wiggins, 2007). DMS is defined by the presence of diffuse mesangial sclerosis in kidney biopsy, with the deeper glomeruli being the least affected. The term DMS relies on the late appearance of the lesion, but does not give any clue on its pathogenesis (Barisoni et al., 2007).

Glomeruli in DMS appear as a halo-shaped distribution of podocytes surrounding a matrix-containing glomerular center (Figure 1). Initially, the matrix has a fibrillary appearance, but at later stages, the mesangial extracellular matrix accumulation becomes more prominent as capillary lumens obliterate with progressive tuft contraction. In parallel, podocytes show absence of foot processes and glomerular capillary loops tend to collapse with progressive podocyte hypertrophy and mild hyperplasia, remaining visible even in advanced disease stages (Barisoni et al., 2007). The tubules are dilated and atrophic, sometimes containing hyaline casts (Charles Jennette et al., 2015). Non-diagnostic deposits of IgM and C3 are seen in the mesangium of the less affected glomeruli and in the periphery of the sclerotic glomeruli (Charles Jennette et al., 2015). Electron microscopy shows mesangial collagen fibrils. The glomerular basement membrane is split and wavy because of zones of subepithelial lucent widening and segmental denudation due to foot process detachment (Charles Jennette et al., 2015).

### Why—Experimental Evidence

**FIGURE 3:**
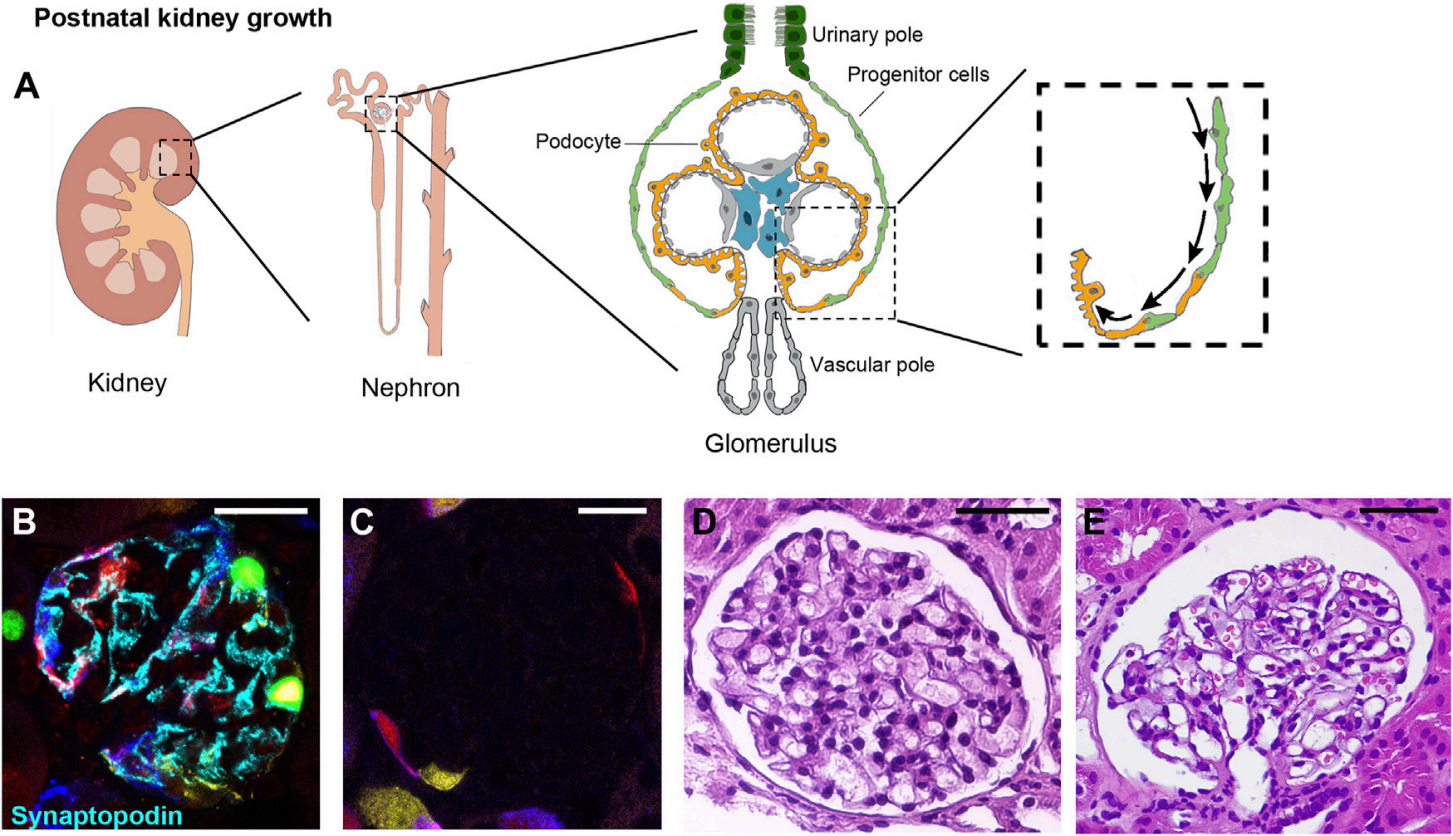
Progenitor cells generate novel podocytes during postnatal kidney growth. **(A)** The kidney is composed of functional units, nephrons, each of which is made of a glomerulus and a tubule. The glomerulus is composed of a tuft of capillaries covered by visceral epithelial cells, the podocytes, and surrounded by the Bowman capsule lined on the inner surface by flat epithelial cells, parietal epithelial cells (PEC). A subpopulation of PEC localized at the urinary pole is endowed with progenitor characteristics and progressively differentiate into podocytes toward the vascular pole of the glomerulus. This occurs as the kidney grows, during childhood and adolescence in mouse models and in humans. In **(B,C)** representative glomeruli from transgenic *Pax2.rtTA;TetO.Cre;R26.Confetti* mice, an established mouse model of renal progenitor cell lineage tracing. In this model, green, yellow, cyan or red fluorescent protein is randomly expressed by Pax2- expressing cells. Pax2 is expressed by PEC progenitor cells during kidney development but is lost upon their differentiation into mature podocytes in the post-natal kidney (*magnification 63x*)*.* In **(B)** a representative glomerulus of *Pax2.rtTA;TetO.Cre;R26.Confetti* mouse, induced at postnatal day P5 (when the generation of new glomeruli from the metanephric mesenchyme has already ended) for 10 days and tracked until 5 weeks of age. Fluorescent Pax2+ cells are present in the parietal epithelium of the Bowman capsule as well as inside the glomerulus. These intraglomerular Pax2-derived cells expressed synaptopodin (cyan), demonstrating their podocyte nature. In (C) a representative glomerulus of a *Pax2.rtTA;TetO.Cre;R26.Confetti* adult mouse induced at 5 weeks of age for 10 days, showing Pax2+ cells only in Bowman capsule. Podocytes are not labeled. **(D,E)** In humans, the observation that the number of podocytes increases during glomerular growth and maturation in the early years after birth, suggest the involvement of a podocyte progenitor pool during postnatal kidney growth. In D a glomerulus of a 4 years old normal human kidney and in E a glomerulus of a 25 years old normal human kidney from two patients who underwent nephrectomy for localized renal tumor (Hematoxylin and eosin stain, magnification 40 x. Bars= 20 μm in B and C, bars= 50 μm in D and E).

Genetic mutations causing DMS have given an insight in podocyte biology. For instance, truncating mutations in phospholipase C epsilon (PLCε1) expressed by podocytes in the developing glomerulus; mutations in WT1, a major podocyte transcription factor present early in podocyte development, or mutations in b2laminin (LAMB2) gene involved in glomerular basement membrane expansion and assembly, are associated with the DMS pattern of injury in humans (Boyer et al., 2010; Lehnhardt et al., 2015; Matejas et al., 2010) and in mouse models (Atchison et al., 2020; Ratelade et al., 2010; Lin et al., 2018). These mutations result in arrested development of glomeruli (Hinkes et al., 2006; Yang et al., 1999) that are not able to prevent protein leakage while filtering, causing massive proteinuria responsible for fast podocyte loss and rapid progression to end stage kidney disease. In particular, glomeruli in kidneys of patients with DMS linked to WT1 mutations show a relatively less-differentiated podocyte phenotype and immunostaining reveals increased expression of the PEC progenitor marker Pax2 (Yang et al., 1999), suggesting migration and attempt of PECs to differentiate into podocytes. Interestingly, gene expression analysis of isolated glomeruli of DMS mouse model obtained introducing a heterozygous WT1 mutation, identified increased expression of Cyp26a1 in podocytes of mutated mice (Ratelade et al., 2010). This resulted in a decrease of the concentration of *all-trans-*retinoic acid—an inducer of PEC progenitor differentiation into podocytes (Peired et al., 2013; Zhang et al., 2012; Dai et al., 2017)—in the glomerular milieu, supporting the hypothesis that a hampered differentiation of PEC progenitors into podocytes could be involved in the development of DMS. In adult animals, mesangial expansion is the first indirect evidence of podocyte loss up to 20% of the podocyte compartment (Wharram et al., 2005). Indeed, in the attempt to maintain the capillary loop open despite the podocyte loss, the mesangium assumes a more prominent fibrillary appearance and expands (Wharram et al., 2005). Reduced number of podocytes, associated with excretion of urinary podocytes, was observed also in glomeruli with severe sclerosis in children with DMS (Ikezumi et al., 2014). Interestingly, some glomeruli showed hypercellular lesions with proliferating cells that stained positive for the PEC marker claudin-1 (Ikezumi et al., 2014), suggesting that podocyte loss and the consequent proliferation of PECs are common processes in the pathogenesis of DMS. Several studies have shown that around 10% of podocytes are formed after birth from PEC progenitors localized at the urinary pole of the Bowman capsule, that progressively differentiate into podocytes, migrating from the urinary pole towards the vascular pole of the glomerulus (Lasagni et al., 2015; Appel et al., 2009; Wanner et al., 2014) (Figure 3). PEC differentiation into podocytes happens substantially to support the increase of kidney dimension as the kidney grows, during childhood and adolescence in mouse models (Lasagni et al., 2015; Appel et al., 2009; Wanner et al., 2014) (Figure 3). Similarly in humans, the finding that podocytes increase in numbers during the first years after birth (Puelles et al., 2015), suggests the involvement of a podocyte progenitor subpopulation driving postnatal glomerular growth (Figure 3). These observations suggest that kidneys from children <5 years are endowed with a higher capacity to generate new podocytes from PECs deriving from the ongoing growth process. This explains why even severe podocyte loss driven by major genetic alterations of the podocyte in young children is associated with a pattern of injury characterized by mesangial expansion and the signs of a massive podocyte turnover. Indeed, in the context of repair attempt by PECs, the initial mesangial adaptive response paralleled by massive introduction of new podocytes along the glomerular basement membrane is enough to prevent scarring and maintain kidney function despite heavy proteinuria in young children (Figure 1 and Table 1). Unfortunately, due to the frequent underlying genetic cause connected to DMS, new podocytes originated by PECs carry the same functional problem of the lost ones, damping the regenerative potential of these patients and progressively leading first to FSGS and then to end stage kidney disease.

## Focal Segmental Glomerulosclerosis

### How—Pathology

The pathognomonic characteristic of FSGS is segmental solidification of the glomerular capillary tuft with hyalinosis and intracapillary foam cells, podocyte hypertrophy and extracellular matrix accumulation in the mesangium, often presented with an adhesion between the capillary tuft and the Bowman capsule (Rosenberg and Kopp, 2017) (Figure 1). Tubulointerstitial scarring is usually associated with glomerular disease and its presence in kidney biopsies with MC lesion suggests FSGS presence on unsampled glomeruli (Rosenberg and Kopp, 2017). Indeed, sampling constitutes an issue in FSGS, as distribution of segmental sclerosis starts in the juxtamedullary glomeruli and progresses towards the outer cortex at later disease stages (Rosenberg and Kopp, 2017). In addition, focality of sclerotic lesions is greater in children than in adults (Fogo, 2015).

Positive staining for IgM and C3 may be revealed by immunofluorescence, and it is believed to represent macromolecular trapping rather than specific deposition (De Vriese et al., 2021). On ultrastructural analysis, electron-dense material may be found in the mesangium and in the subendothelial compartment, consistent with hyalinosis (Barisoni et al., 2007).

FSGS is a pattern of injury shared by different diseases with variable clinical courses. To address this heterogeneity, the first attempt to classify FSGS relied on pathologic presentation describing five variants (D'Agati et al., 2004): the perihilar variant, with FSGS lesion located at the vascular pole; the tip lesion variant, with lesion located at the urinary pole; the cellular variant, characterized by endocapillary hypercellularity; the collapsing variant, characterized by collapse of the capillary tuft with epithelial cell hypertrophy and hyperplasia; and lastly FSGS not otherwise specified if lesions do not fit in the other variants mentioned (D'Agati et al., 2004). Unfortunately, with the exception of the tip lesion variant, which is usually associated with response to steroid treatment, the other variants have not provided sufficient help in patient stratification, mostly because the not otherwise specified variant represents by far the most frequent one. However, the common feature of FSGS is absolute or relative podocyte depletion as also demonstrated by the presence of podocyturia in FSGS patients (Szeto et al., 2015).

### Why—Experimental Evidence

Podocyte injury is a major trigger of glomerulosclerosis but alone may not be sufficient to cause sclerosis as observed in the MC lesion. Additional cellular processes, including podocyte detachment are necessary to reach a critical threshold of podocyte depletion (Wharram et al., 2005), after which glomerulosclerosis occurs. The FSGS lesion is not due to a specific glomerular disease. Indeed, several conditions are well-described causative insults that lead to podocyte depletion such as hyperglycemia and insulin signaling, mechanical stress, angiotensin II, calcium signaling, viral infection, toxins, oxidants, and immunological injury (Zhong et al., 2019). Thus, a wide range of disease states can lead to the development of the FSGS injury pattern, the common denominator being that the initiating events take place in podocytes. FSGS animal models provided the proof-of-concept that podocyte depletion is a major factor mediating proteinuria and glomerulosclerosis. In particular, in the DT model, 21–40% podocyte depletion showed mesangial expansion, capsular adhesions, focal segmental glomerulosclerosis, mild persistent proteinuria and normal renal function, while more than 40% podocyte depletion showed segmental to global glomerulosclerosis with sustained high-grade proteinuria and reduced renal function (Wharram et al., 2005). Nevertheless, following podocyte loss, subsequent local responses are also critical for segmental sclerosis to occur. Indeed, following loss of podocyte coverage due to death or detachment, the uncovered glomerular basement membrane loses structural support by overlying podocytes at these sites (Jefferson and Shankland, 2014). Consequently, the capillary loop may bulge toward the Bowman capsule and an early connection, tuft adhesion, forms between PECs and the uncovered glomerular basement membrane or between podocytes and PECs (Löwen et al., 2021). As a response, PECs become focally activated, *de novo* express the specific markers CD9 and CD44 (Okamoto et al., 2013; Lazareth et al., 2019; Ito et al., 2020), migrate to a visceral location and deposit matrix. Interestingly, the CD44 marker is scarcely expressed by PECs in human MC lesion and may represent a useful marker to distinguish the MC and FSGS lesions in human biopsies (Smeets et al., 2014; Kuppe et al., 2015).

Several studies suggested that the presence of PECs on the glomerular tuft represents an attempt to replenish the podocyte pool (Romoli et al., 2018; Lasagni et al., 2015; Peired et al., 2013; Kaverina et al., 2019; Eng et al., 2015; Zhang et al., 2013; Kaverina et al., 2020). The capacity of a PEC subset to differentiate into podocytes and restore the podocyte number is the likely explanation for the clinical observations of remission and regression of functional parameters of chronic kidney disease in patients with diabetic and nondiabetic nephropathies (Cortinovis et al., 2016; Fioretto et al., 1998; Remuzzi et al., 2006; Takahashi et al., 2007), as well as for podocyte number restoration observed in response to pharmacological treatment in experimental models of diabetic and non-diabetic kidney disease (Zhang et al., 2012; Hudkins et al., 2020; Zhang et al., 2015). Recent results elucidated the podocyte-progenitor cross-talk revealing mediators of progenitor quiescence during homeostasis and mechanisms by which podocyte loss triggers the activation of the progenitor population in the setting of podocyte injury (Peired et al., 2021). In addition, they also provide possible explanation of why in certain conditions podocyte replacement by PECs may not be successful and lead to development of the FSGS lesion (Peired et al., 2021). In healthy conditions, the constitutive production of CXCL12 by podocytes maintains local podocyte progenitors in a quiescent state (Romoli et al., 2018), while the reduced expression of CXCL12 consequent to podocyte loss, promotes activation, migration and differentiation of PECs into podocytes (Romoli et al., 2018). Podocyte loss also permits the passage through the damaged glomerular filtration barrier of circulating retinol that is subsequently transformed into the Bowman space in retinoic acid that acts as an inducer of progenitor differentiation into podocytes (Peired et al., 2013; Zhang et al., 2012; Dai et al., 2017). The availability of retinoic acid for PECs is strongly reduced in the presence of a high-grade proteinuria, due to the retinoic acid sequestration operated by albumin in the Bowman space (Peired et al., 2013), with a consequent inefficient progenitor-to-podocyte differentiation. This observation likely explains the well-known clinical observation that high proteinuria associates with FSGS progression, while reduction of proteinuria by renin-angiotensin system blockers retards progression and restores podocyte number (Fogo, 2015; Boffa et al., 2003; Ma et al., 2005). Interestingly, increasing progenitor responsiveness to retinoic acid signaling through pharmacological approaches, such as administration of 6-bromo-indirubin-3′-oxime, mitigates glomerulosclerosis progression in non-diabetic (Lasagni et al., 2015) and diabetic mouse models (Motrapu et al., 2020), demonstrating that the progenitor-to-podocyte axis is a potential therapeutic target. PEC differentiation into podocytes appears strongly dependent on mechanical conditions present inside the glomerulus, such as its stiffness (Melica et al., 2019), that changes during the course of several diseases (Embry et al., 2018; Wyss et al., 2011) and precedes the appearance of glomerular sclerosis, as observed in the early phase of a HIV-associated nephropathy model (Embry et al., 2018). In conclusion, the FSGS pattern represents the pathology expression of a scar generated by a chronic podocyte loss (Motrapu et al., 2020) that exceeds the capacity for podocyte replacement provided by PECs, either because the podocyte loss is severe or because the capacity of PECs to differentiate into podocytes is hampered by an excessive proteinuria or an altered glomerular basement membrane stiffness (Table 1). Interestingly, juxtamedullary nephrons that show low numbers of PECs with progenitor capacity are particularly sensitive to development of sclerosis (Romoli et al., 2018) while superficial and mid-cortical nephrons harbor a higher number of podocyte progenitors, explaining the reported increased regenerative capacity of these glomeruli, and their resistance to development of FSGS lesions (Romoli et al., 2018). However, when podocyte loss overcomes PEC capacity of regeneration, glomerular basement membrane denudation occurs followed by synechia formation and ultimately sclerosis resulting in FSGS pattern (Figure 1).

## Collapsing Glomerulopathy

### How—Pathology

CG is a pathology pattern characterized by the presence of segmental capillary tuft collapse (wrinkling and folding) in at least one glomerulus, in association with podocyte hypertrophy and/or hyperplasia (D'Agati et al., 2004). The Columbia classification had the merit to recognize the collapsing pattern as part of the same family of FSGS (D'Agati et al., 2004). Afterwards, terminology has evolved in collapsing glomerulopathy to underline the rapid and catastrophic collapsing nature of the pathological process. CG is histologically defined by the formation of a pseudocrescent, i.e., a massive proliferation of cuboidal undifferentiated epithelial cell in the Bowman space, leading to a collapse of the capillary loops, regardless of the extracellular matrix accumulation eventually leading to focal and global glomerulosclerosis (Muehlig et al., 2021) (Figure 1). This pattern of injury represents a common endpoint from multiple etiologies (APOL1 risk variants, infections, drugs, ischemia, hematologic neoplasia and autoimmune disease), suggesting a common pathological mechanism rather than a specific cause (Nicholas Cossey et al., 2017).

### Why—Experimental Evidence

Immunohistochemistry and ultrastructural studies suggested that a primary damage to podocytes alone is sufficient to initiate the events underlying the formation of pseudocrescents (Henderson et al., 2008). Although the swollen and proliferating abnormal cells within the Bowman space involved in pseudocrescent formation lacked the expression of podocyte markers, they were previously regarded as “dysregulated or dedifferentiated” podocytes that had re-entered the cell cycle to proliferate (Barisoni et al., 1999). This assumption was mostly based on their occasional positivity for the cell cycle marker Ki67. However, positivity for Ki67 indicates cell cycle entry and not necessarily mitosis (Lazzeri et al., 2019). Indeed, after injury, podocytes can re-enter the S phase of the cell cycle (Frank et al., 2022) to undergo hypertrophy, and stain for Ki67 (Marshall and Shankland, 2006). However, if they are forced to bypass the G2/M cell cycle checkpoint, podocytes undergo aberrant mitosis and consequent detachment or death for mitotic catastrophe (Barisoni et al., 2000; Suzuki et al., 2009; Lasagni et al., 2013; Liapis et al., 2013; Al Hussain et al., 2017). Thus, consistent with their status of highly differentiated post-mitotic cells, staining for cell cycle markers in podocytes should not be interpreted as a sign of mitosis. On the contrary, it may be suggestive of an endoreplication process as shown in multiple organs (Lazzeri et al., 2019; Bischof et al., 2021).

A typical clinical condition associated with collapsing pattern of injury at kidney biopsy is viral-mediated nephropathy, such as that observed with HIV, Parvovirus and SARS-CoV-2 infection (Muehlig et al., 2021). Pseudocrescents, collapse of the capillary loops and podocyte multinucleation are predominant features of HIV-associated nephropathy (Barisoni et al., 2000; Suzuki et al., 2009; Lasagni et al., 2013; Liapis et al., 2013; Al Hussain et al., 2017). Indeed, virus-induced podocyte mitosis is catastrophic and induces podocyte death. In addition, immunohistochemical studies in idiopathic, HIV-associated, and pamidronate-associated CG have shown that cells comprising the pseudocrescents in human biopsies express proteins characteristic of PECs, such as cytokeratin, Pax2, CD24, and specific glycosylated isoform of CD133 (glycCD133), suggesting that cells within the pseudocrescents have a parietal epithelial rather than podocyte origin (Dijkman et al., 2005; Dijkman et al., 2006; Smeets et al., 2009). Lineage tracing by genetic tagging employing both podocyte and PEC-specific reporter mice in a model mimicking CG finally proved that hyperplastic cells were not podocyte-derived, but of PEC origin (Suzuki et al., 2009). Moreover, a recent multiomics study reported that podocyte-specific knockdown of Krüppel-like factor 4 contributes to podocyte loss triggering the activation of a distinct PEC subpopulation, suggesting that in this disorder PEC proliferation and pseudocrescent formation represent a response to podocyte injury and loss (Pace et al., 2021).

Experimental evidence demonstrated that, collapsing nephropathy and pseudocrescents originate from the proliferation of a specific PEC subpopulation expressing CD133 and Pax2 markers and representing renal progenitor cells that abnormally shift their reactions from reparative to detrimental (Yang et al., 2002; Smeets et al., 2004). It is unclear which factors are responsible for tilting the balance. It was proposed that pseudocrescents originate from PEC progenitors as a dysregulated response to the massive and fast podocyte detachment occurring in conditions of direct podocyte injury caused by drugs exposure, immune-mediated disorders or viral infections that cause a fast, massive podocyte loss leading to capillary collapse (Al Hussain et al., 2017; Kopp et al., 2020). In viral glomerulopathies, type I interferons (IFNs) are important mediators of viral infection (Anders et al., 2010). Indeed, in the biopsy of a patient with monogenic type I interferonopathy, MxA, a protein involved in antiviral immunity and induced by type I IFNs, was selectively expressed in CD133 positive PECs but not in podocytes (Fenaroli et al., 2021). *In vivo*, in a model of Adriamycin nephropathy, the injection of either IFN-α and IFN-β aggravated proteinuria and glomerulosclerosis and correlated not only with the triggering of local inflammation inside the glomerulus but also with a direct effect on podocytes and PECs (Migliorini et al., 2013). IFN- β specifically promoted podocyte loss by inducing mitotic catastrophe in podocytes (Migliorini et al., 2013). IFN- α affected PEC proliferation and migration (Migliorini et al., 2013). Both IFNs also impaired the differentiation of renal progenitors into mature podocytes, a mechanism that favors focal scarring over glomerular repair (Migliorini et al., 2013). Collapsing glomerulopathy has also been described in patients receiving exogenous IFN therapy administered for various medical conditions (Markowitz et al., 2010), further confirming that IFNs are critical mediators of the collapsing pattern of injury. Moreover, a growing body of evidence supports the role of IFNs as inducers of CG in individuals with the APOL1 high-risk genotype (Abid et al., 2020). APOL1 risk variants G1 and G2 are known to result in risk for kidney disease in patients of African ancestry and associate with a heterogeneous pattern of injury. Collapsing glomerulopathy is the most fulminant pattern of injury associated with APOL1-nephropathy (Abid et al., 2020). This form of glomerulopathy is observed mostly in diseases that have increased IFN levels, such as HIV infection and systemic lupus erythematosus (Larsen et al., 2013; Abid et al., 2020; Goyal and Singhal, 2021). APOL1 regulates PEC molecular phenotype through modulation of miR-193a expression through reciprocal feedback (Kumar et al., 2018). Indeed, PEC differentiation into podocytes is accompanied by a decrease in miR-193a expression (Kietzmann et al., 2015). Similarly, the suppression of miR-193a enhances APOL1 expression (Jessee and Kopp, 2018). Taken altogether, these observations suggest that the expression of APOL1 in PECs contributes to their differentiation into podocytes and the absence of APOL1 promotes PEC phenotype maintenance (Kumar et al., 2018). These data support the hypothesis that in the presence of massive and fast podocyte detachment observed in the collapsing pattern of injury, APOL1 risk variants aggravate the clinical outcome by hampering *de novo* formation of podocytes. Rapid and massive podocyte loss does not allow a mesangial adaptive response and abruptly stimulates the podocyte-progenitor feedback (Kietzmann et al., 2015; Jessee and Kopp, 2018; Kumar et al., 2018; Goyal and Singhal, 2021). The PEC compartment responds promptly with proliferation but it fails to complete the differentiation process towards mature podocytes. This results in obliteration of Bowman capsule with immature elements further compressing the glomerular tuft that lacks support from external and internal sides (Figure 1 and Table 1).

## Conclusions

Altogether, these observations suggest that podocytopathies represent a complex group of disorders of the glomerular epithelial compartment, where the equilibrium between the nature, the length and the severity of podocyte injury as well as the efficiency of the repair response provided by PECs ultimately determines the pattern of injury observed at the biopsy as well as renal prognosis. A variety of genetic variants contributes to both podocyte injury and PEC repair response affecting kidney disease progression. Standard pathology techniques are not able to identify the ongoing evolution of these alterations but merely show the histological appearance that results from the process. In MC lesion only the slit diaphragm is damaged, and the structural alterations are reversible, either because the podocyte is not lost, or because PECs succeed in differentiating into new podocytes and maintaining full coverage of the filtration barrier. In FSGS, the balance between podocyte injury and replacement is lost, triggering a vicious circle where proteinuria prevents PEC progenitor cells from appropriately facing podocyte loss. In contrast, if PECs succeed in generating new podocytes, scar formation can be contained and limited to a certain extent. However, this high regenerative potential is restricted to a specific and relatively short age span, explaining why DMS is observed only in children. Finally, a fast and massive podocyte loss determining the collapse of the glomerular capillary loops is the key mechanism of CG. The ability of PECs to proliferate is retained, but the capacity to differentiate into mature podocytes is prevented, causing massive PEC activation, ultimately resulting in pseudocrescents that are typical of CG. Understanding the molecular and cellular alterations that lead to the generation of these patterns of injury can help the clinicians to convey the right diagnosis and the researchers to identify novel potential therapeutic targets for podocytopathies.
